# The Challenges of Implementing Good Health and Well-Being During a Pandemic: A Case Study of the Behavior of Using Telemedicine Services in the Younger Generation

**DOI:** 10.1155/2024/4561336

**Published:** 2024-11-04

**Authors:** Endang Parahyanti, Afiyah Tsarwat Zharifah, Sinan Vidi Lazuardi

**Affiliations:** ^1^Faculty of Psychology, Universitas Indonesia, Depok 16424, Indonesia; ^2^Research Cluster of Interaction, Community Engagement, and Social Environment, Universitas Indonesia, Central Jakarta 10430, Indonesia

**Keywords:** behavioral pattern, health service, pandemic, telemedicine, young generation

## Abstract

**Objectives:** Telemedicine has emerged as a crucial tool in addressing public health requirements, particularly during a pandemic. This aligns with the third Sustainable Development Goals (SDGs) objective of ensuring healthy lives and promoting well-being for all ages. The current generation demonstrates a greater proficiency in modern technology, prompting researchers to investigate their views and behavior trends in telemedicine.

**Methods:** This research is aimed at examining the telemedicine adoption patterns of Generation Y and Generation Z by dividing them into two groups. Study Group 1 is comprised of those who have known modern technology but have yet to gain experience in using telemedicine. Meanwhile, Study Group 2 is comprised of those who had used telemedicine services. Utilizing structural equation model (SEM) analysis, this research investigates the magnitude of influence exerted by various determinants on an individual's tendency to utilize telemedicine.

**Results:** Findings from both study groups indicate that younger generations have positive attitudes towards telemedicine, significantly influencing their willingness to use telemedicine. Furthermore, the level of readiness for change emerges as a crucial factor in determining the degree of affective commitment to change, continuance commitment to change, and normative commitment to change. In addition, the participants' affective experience state significantly influences all three dimensions of commitment to change.

**Conclusion:** The results suggest the importance of fostering a positive and pragmatic understanding of telemedicine among young people. This can be achieved by encouraging the use of telemedicine services and fully adopting the shift to telemedicine. Therefore, it is crucial to prioritize the development of a positive view towards telemedicine, provide excellent service experiences, and promote social flexibility to attract a large user base, especially among the younger population.

## 1. Introduction

The background of this research will be discussed in this section. The COVID-19 pandemic has occurred since 2020. COVID-19 is a disease driving global health challenges and economic disasters. This public health crisis has also developed other problems for society, such as mental health problems, which are a driving factor in improving the healthcare system [1]. Meanwhile, the response to this pandemic depends on the country's health system and universal health coverage, including the preparedness of the public health system [2]. Various approaches from diverse fields, including clinical science, medical science, public health, sociology, social psychology, and health economics, have emerged to find solutions for fighting the COVID-19 virus and transitioning to a “new normal” era [3, 4]. One critical strategy to prevent the spread of the virus is to encourage the public to forgo in-person visits to hospitals and doctors' offices in favor of telemedicine consultations. The COVID-19 pandemic telemedicine services strive for remote communication between patients and healthcare workers by ensuring the provision of cost-effective, convenient, and timely healthcare services for everyone with a secure communication process using virtual clinics via text, phone call, email, video-based conference, or healthcare applications [5, 6].

Most countries around the world have been implementing telemedicine in recent years, with some countries having a long history of utilizing this technology. The pandemic has undeniably brought unprecedented challenges to how medical centers treat their patients. However, it is essential to consider and define the role of telemedicine beyond the context of a pandemic and postpandemic [7, 8]. Since the previous pandemic, video consultations between residents have significantly increased. Telemedicine allows people to access their medical needs remotely, including healthcare information and services. Several elements of long-term telemedicine sustainability include developing a skilled workforce, empowering consumers, funding reform, enhancing the digital ecosystem, and integrating telemedicine into routine care delivery [9]. These requirements highlight the need for integration between stakeholders to ensure the natural implementation of telemedicine. A previous study has also mentioned that a multistakeholder approach is essential to overcome the digital divide and unequal access to telemedicine [10].

Telemedicine implementation in communities is determined by payment constraints, provider concerns, and organizational barriers [8]. Research conducted by Rahi, Khan, and Alghizzawi [11] also further identified that the adoption of telemedicine in society is influenced by expectancy, facilitating conditions, habit, hedonic motivation, price values, information quality, system quality, and service quality. Additionally, Rahi's study revealed that the utilization of telemedicine is more pronounced when individuals perceive a higher severity of their illness. Patients expressed a greater optimism regarding the potential of telemedicine compared to their counterparts in private practices [12]. The rise of telemedicine reflects the rapid advancement of innovative technologies and is becoming an essential tool for healthcare teams worldwide. This innovation was quickly deemed necessary as it facilitated communication between healthcare workers and geographically isolated patient populations. Telemedicine also addresses the limitations of regional healthcare infrastructure and service providers, offering time and resource efficiencies for all parties, such as health workers and patients [13]. These facts also imply that telemedicine is becoming an integral component of the evolving healthcare landscape.

While several digital providers and the government have collaborated in developing telemedicine services, the people's readiness to adopt this technology must be ensured. In general, implementing telemedicine itself is not easy. Research suggests that the failure rate in implementing telemedicine is 70%. One of the main factors is the lack of readiness to adopt the technology [14]. Moreover, the landscape of telemedicine services is predominantly established by startups rather than hospitals, even though digital health services from hospitals enjoy higher public trust due to their perceived robust ecosystem [15]. While telemedicine offers many benefits within the healthcare field, it is essential to acknowledge that it may not provide the same level of service as face-to-face healthcare encounters and does not facilitate them. Furthermore, despite the Health Information Technology for Economic and Clinical Health (HITECH) Act of 2009 permitting the transfer of protected health information through electronic systems, there are significant risks associated with privacy breaches [13]. Furthermore, telemedicine implementation faces several challenges, including high prices, immature infrastructure, confidentiality concerns of sensitive health data, and potential resistance to change [16]. In other words, the application of telemedicine in healthcare services has advantages and disadvantages because of the significant changes that everyone must face. Therefore, consumer attitudes must be understood, as technological advancement that is not driven by a positive attitude may not be well received [17].

Understanding the factors influencing telemedicine adoption behavior is crucial for the healthcare and digital technology industry [17]. Hoque and Sorwar [18] demonstrated that performance expectancy, effort expectancy, social influence, technology anxiety, and resistance to change significantly influenced the users' behavioral intention. As the postpandemic landscape normalizes telemedicine as a standard component of healthcare, it is critical to examine the path towards making it a foundational element of the care cycle instead of just a luxury [19]. In this study, we will explore the behavioral patterns of the younger generation when using telemedicine services. The younger generation is chosen as they dominate today's population and are more familiar with digital solutions for various needs. The younger generation's awareness of digital technology is already high, especially in urban areas [11]. However, while telemedicine has gained acceptance in all professions, its implementation in different care scenarios is confronted with a variety of different challenges. In planning and maintaining telemedicine, readiness assessment can be a valuable tool to improve the chances of successful implementation by identifying the stakeholders and the factors that should be targeted [20]. The term readiness embraces preparedness, receptiveness, and the willingness to achieve successful implementation [20].

## 2. Research Contribution

The contribution of this research will be discussed thoroughly in this section. Previous studies have predominantly focused on the readiness of health workers and telemedicine providers. However, a critical gap regarding the readiness of society to become telemedicine users emerges. Therefore, this study is aimed at discovering user readiness for telemedicine adoption, specifically among younger generations. This focus will be a novelty in research pertaining to telemedicine topics. This study explores the key elements that significantly contribute to the readiness for change, intention, experiences, and commitment to using telemedicine among young generations. By integrating the theory of planned behavior (TPB) to examine the factors shaping young generations' intention to adopt telemedicine and social exchange theory to examine the factors influencing their commitment to sustained telemedicine use, this research will enhance the existing literature and offer new insights into young generations' intentions to adopt telemedicine, as well as their readiness and commitment to shift from traditional healthcare services to telemedicine.

## 3. Organizational Structure of This Research

In this section, the organizational structure of this research will be discussed. This research is structured as follows: (1) Introduction section that provides background information and contribution of the research, (2) Materials and Methods section that details the research design, hypothesis development from Study 1 and Study 2, and the statistical analysis employed in this research, (3) Results section that consists of both Study 1 and Study 2 instrumentation and data analysis result, (4) Discussion section that discusses the result from both Study 1 and Study 2 and a comparison to previous research, (5) Conclusions section that concludes the result of both of Study 1 and Study 2 result, theoretical implication, and managerial implication, and (6) Limitation and Future Research section that describes the limitation of this study and aspects that should be considered in the future studies.

## 4. Materials and Methods

### 4.1. Study Design and Sampling

The study design and sampling used in this study will be discussed in this section. This research was based on a quantitative approach. Quantitative research involves instrument-based questions, statistical analysis, and statistical interpretation [21]. It typically begins with established hypotheses and theories and employs experimental or manipulative approaches using experiments, surveys, quantitative content analysis, structured interviews, and others [22]. The sampling technique used was purposive sampling, where participants are selected based on their perceived ability to provide information most relevant to the research objectives [23].

This study's participants are Generation Y and Generation Z. Generation Y encompasses individuals aged 23–40, and Generation Z refers to individuals aged 18–22 [24]. Therefore, this study uses the age range from < 18 to 40 years old. In addition, the young generation participants were chosen as they reside in Surabaya, once one of the cities with the highest COVID-19 death cases in Indonesia. Then, participants were divided into two groups: Study 1, the young generation who had never used telemedicine, and Study 2, the young generation who had used telemedicine.

Data collection for this study employed a survey instrument in the form of a questionnaire distributed to young adults residing in Surabaya, Indonesia, via Google Forms. The questionnaire included a screening question: “Do you know what telemedicine services are?” to sort out the young people who are familiar and unfamiliar with telemedicine. Following this initial screen, Study 1 included 138 young adults aware of telemedicine services, while Study 2 included 170 participants with prior telemedicine experience.

This study aligns with the positivist paradigm in research methodology. Positivism is a paradigm that relies on the deductive method, often stated quantitatively, and involves causal and explanatory factors; the quantitative approach is mainly based on the positivist paradigm. The positivist paradigm asserts that knowledge can be attained through experiments and unbiased empirical observations conducted via meticulous scientific methods [25]. Positivism has been involved in making hypotheses, operationalizing variables, and conducting empirical studies [26]. This study takes direct measurements and employs systematic procedures by administering questionnaires. The data collected will then be subjected to quantitative analysis utilizing statistical techniques such as structural equation modeling (SEM) and confirmatory factor analysis (CFA). This methodological approach allows the research to empirically test hypotheses and gain a deeper understanding of the cause-and-effect relationships associated with telemedicine service utilization among young generations. Therefore, from the procedures and methods mentioned, this study uses a quantitative approach that relies on a positivist paradigm.

### 4.2. Study 1: The Young Generation Who Had Not Used Telemedicine

Study 1 will examine the readiness for change, commitment to change, and intention to use telemedicine among individuals who have yet to utilize telemedicine. Individuals' commitment is considered a crucial factor in supporting a successful change [27]. Commitment to change consists of affective commitment to change, ongoing commitment to change, and normative commitment to change. Readiness for change can positively impact each of these components [28]. Previous research shows the importance of readiness to change in the transition process of social change. A supportive environment, such as collaboration between parties in socializing change, can also trigger commitment to change [29, 30]. In the context of telemedicine, other strategies, such as promoting telemedicine, can also be carried out on social media to increase public interest in adopting this healthcare behavior. Therefore, readiness to change is essential to the younger generation's transition to digital healthcare technology. This study assumed that the readiness for change of the younger generation will also play a role in each commitment to change, especially in the context of shifting from traditional healthcare services to telemedicine. In addition, research by [31] also commits a determining factor for individual behavioral intentions, suggesting a positive relationship between commitment and intention.

**Figure 1:**
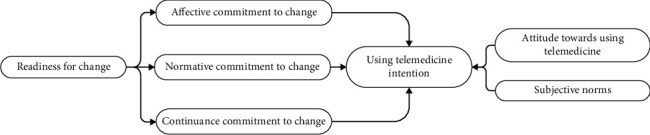
Study 1 research model.

Furthermore, behavioral intentions are generally explained by the TPB [32]. Several factors influence an individual's behavioral intention to engage in a specific behavior, which in this study means using telemedicine. These factors include attitude towards behavior, subjective norms, and perceived behavioral control. However, another research found that perceived behavioral control did not play a significant role in the intention to use a certain application [33]. Based on this finding, perceived behavioral control will not be included as a variable in this study. Therefore, the research hypothesis in Study 1 is as follows (see Figure 1).

**Hypothesis 1 .**

Readiness for change contributes to affective commitment to change among younger generations who have never used telemedicine.

**Hypothesis 2 .**

Readiness for change contributes to continuance commitment to change among younger generations who have never used telemedicine.

**Hypothesis 3 .**

Readiness for change contributes to normative commitment to change among younger generations who have never used telemedicine.

**Hypothesis 4 .**

Affective commitment to change plays a role in the intention to use telemedicine among younger generations who have never used telemedicine.

**Hypothesis 5 .**

Continuance commitment to change plays a role in the intention to use telemedicine among younger generations who have never used telemedicine.

**Hypothesis 6 .**

Normative commitment to change plays a role in the intention to use telemedicine among younger generations who have never used telemedicine.

**Hypothesis 7 .**

Attitude towards using telemedicine plays a role in the intention to use telemedicine among younger generations who have never used telemedicine.

**Hypothesis 8 .**

Subjective norms play a role in the intention to use telemedicine among younger generations who have never used telemedicine.

### 4.3. Study 2: The Young Generation Who Had Used Telemedicine

Study 2 will examine the readiness for change, commitment to change, and customer experience among individuals who have previously utilized telemedicine. The younger generation who has used telemedicine responds differently than those who have never used telemedicine. However, commitment to change remains a crucial factor in the success of these changes [34]. This is because commitment to change reflects a mindset that binds a person to a new approach [35]. Commitment also serves as a measure of the relationship between the customer and the goods/service provider, which is often shaped by the customer's experience [36]. The more positive the experience individuals have when using telemedicine, the more it will shape their loyalty to telemedicine in the future [37].

**Figure 2:**
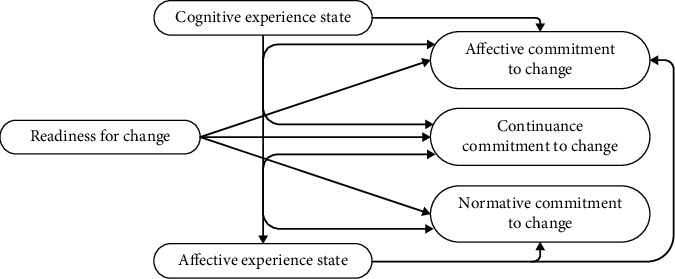
Study 2 research model.

Telemedicine is the use of a service conducted online, so the experience is called an online customer experience. Online customer experience can be categorized into two psychological aspects, namely, cognitive experiential states and affective experiential states [38]. Therefore, this study posits that online customer experience, in terms of both cognitive experience state and affective experience state, can play a role in the commitment to change. This study will also explore how they perceive the use of telemedicine itself. These insights can serve as a valuable input for telemedicine service providers in Indonesia to improve their quality. Therefore, the research hypothesis in Study 2 is as follows (see Figure 2).

**Hypothesis 9 .**

Readiness for change contributes to affective commitment to change among younger generations who have used telemedicine.

**Hypothesis 10 .**

Readiness for change contributes to continuance commitment to change among younger generations who have used telemedicine.

**Hypothesis 11 .**

Readiness for change contributes to normative commitment to change among younger generations who have used telemedicine.

**Hypothesis 12 .**

Cognitive experience state plays a role in affective commitment to change among younger generations who have used telemedicine.

**Hypothesis 13 .**

Cognitive experience state plays a role in continuance commitment to change among younger generations who have used telemedicine.

**Hypothesis 14 .**

Cognitive experience state plays a role in normative commitment to change among younger generations who have used telemedicine.

**Hypothesis 15 .**

Affective experience state plays a role in affective commitment to change among younger generations who have used telemedicine.

**Hypothesis 16 .**

Affective experience state plays a role in continuance commitment to change among younger generations who have used telemedicine.

**Hypothesis 17 .**

Affective experience state plays a role in normative commitment to change among younger generations who have used telemedicine.

## 5. Results

### 5.1. Study 1

#### 5.1.1. Demography

In this section, the demography of the participants in the study will be described. In Study 1, it was found that 138 young people had never used telemedicine. The sample of young people in this study consisted of men (*n* = 39; 28.26%) and women (*n* = 99; 71.74%); aged < 18 years (*n* = 7; 5.07%), 18–30 years (*n* = 121; 87.68%), and 31–40 years (*n* = 10, 7.25%); and had a high school education (*n* = 124; 89.86%), bachelor's degree (*n* = 13; 9.42%, and master's degree (*n* = 1; 0.72%).

#### 5.1.2. Measurement

The measurement of the instrument for SEM analysis will be discussed in this section. Before conducting the SEM test, the instruments underwent a CFA test to minimize the potential of eliminating too many items during the SEM test and to get the goodness of fit for each instrument as a validity test. In addition, a reliability test with Cronbach's alpha (∝) was also conducted. This instrument test was conducted using JASP 0.16.1.0. Six out of the seven instruments identified at least one item that detracted from achieving an acceptable fit model, except for intensive telemedicine. Thus, the data shown in Table 1 were obtained.

#### 5.1.3. Data Analysis and Results

This section explains the data analysis and results in Study 1. This data analysis has assessed participants' readiness for change, intention, and commitment to change when using telemedicine. Additionally, this analysis also explored the connection between each of those variables. The data analysis techniques used in Study 1 were descriptive analysis and SEM test. A descriptive analysis was conducted using Microsoft Office Excel to see the demographic distribution of young generation participants, including gender, age, occupation, and education level. Furthermore, the SEM test was used to analyze the relationship patterns between variables and the measurement indicators of each variable in one model.

**Figure 3:**
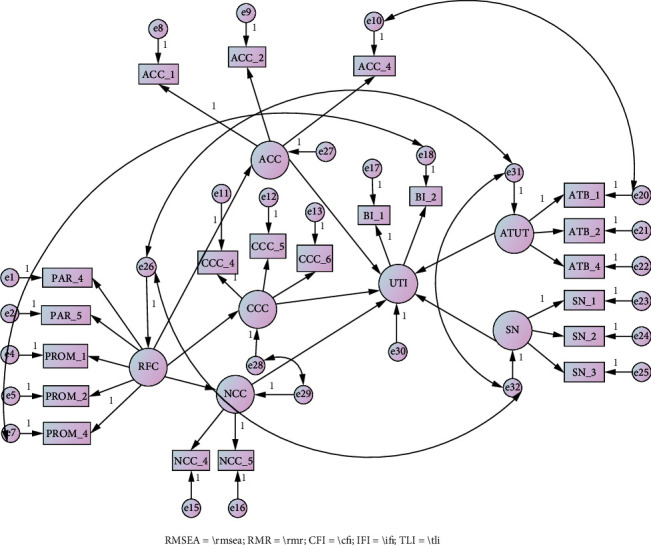
Results of data analysis from Study 1. RFC = readiness for change; ACC = affective commitment to change; CCC = continuance commitment to change; NCC = normative commitment to change; UTI = using telemedicine intention; ATUT = attitude towards using telemedicine; SN = subjective norms.

Based on the SEM test that has been conducted, several variables and items need to be correlated to get a fit model. The correlated variables include readiness for change with attitude towards using telemedicine and subjective norms, attitude towards using telemedicine with subjective norms, and continuance commitment to change with a normative commitment to change. In addition, several items need to be eliminated in the readiness for change construct, namely, one from the participating dimension and one from the promoting dimension. Similarly, one item was eliminated from both the construct of normative commitment to change and telemedicine intention. Finally, the SEM model was fit with RMSEA = 0.061, RMR = 0.037, CFI = 0.953, IFI = 0.954, and TLI = 0.943. Furthermore, for hypothesis testing, the results of the AMOS 23 output are shown in Figure 3.

The results presented in Study 1 show that readiness for change plays a role in all components of commitment to change, namely, affective commitment to change, continuance commitment to change, and normative commitment to change. In addition, attitude towards change plays a role in using telemedicine intention. However, it was found that all components of commitment to change and subjective norms did not play a role in using telemedicine intention (see Table 2).

### 5.2. Study 2

#### 5.2.1. Demography

This section will describe the demography of participants in Study 2. In Study 2, it was found that 170 young people had used telemedicine. The sample of young people in this study consisted of men (*n* = 47; 27.65%) and women (*n* = 123; 72.35%); aged < 18 years (*n* = 10; 5.88%), 18–30 years (*n* = 149; 87.65%), and 31–40 years (*n* = 11, 6.47%); and had a high school education (*n* = 152, 89.41%) and bachelor's degree (*n* = 18, 10.59%). In addition to the demographics of the participants, Study 2 also obtained an overview of the activities conducted on telemedicine applications commonly used by these young generation participants, which consisted of consulting a doctor (*n* = 85; 50%), reading articles (*n* = 55; 32.35%), buying medicines (*n* = 19; 11.18%), ordering laboratory services (*n* = 2; 1.18%), getting COVID-19 drugs (*n* = 2; 1.18%), and others (*n* = 7; 4.11%).

#### 5.2.2. Measurement

This section explains the measurement for using SEM analysis in Study 2. Before conducting the SEM test, the instruments underwent a CFA test to minimize the potential of eliminating too many items during the SEM test and to get the goodness of fit for each instrument as a validity test. In addition, a reliability test with Cronbach's alpha (∝) was also conducted. This instrument test was conducted using JASP 0.16.1.0. Four out of the six instruments identified at least one item that detracted from achieving an acceptable fit model, except for the cognitive state. Thus, the data shown in Table 3 were obtained. The instruments used to describe the perceptions and experiences of respondents are in the form of open questions that are aimed at exploring the perceptions and experiences of young people who have used telemedicine. The questions included their experience using telemedicine, what they liked and did not like about it, and their future expectations regarding telemedicine services.

#### 5.2.3. Data Analysis and Results

This section explains the data analysis and results in Study 2. This data analysis has assessed participants' readiness for change, experience, and commitment to change when using telemedicine. Additionally, this analysis also explored the connection between each of those variables. The data analysis techniques used in Study 2 were descriptive analysis and SEM test. Descriptive analysis was conducted using Microsoft Office Excel to see the demographic distribution of young generation participants, including gender, age, occupation, latest education, and the commonly conducted activities on telemedicine applications. Furthermore, the SEM test was conducted to analyze the relationship patterns between variables and each variable's measurement indicators in one model. In addition, data from open-ended questions were also analyzed using percentages.

**Figure 4:**
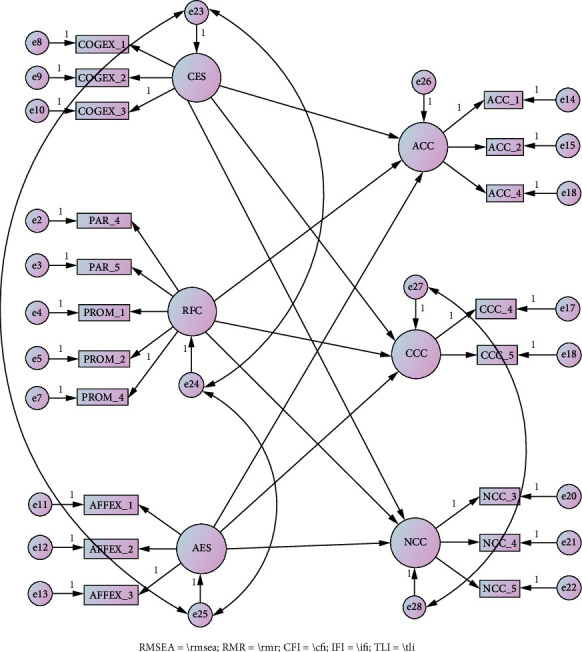
Results of data analysis from Study 2. RFC = readiness for change; ACC = affective commitment to change; CCC = continuance commitment to change; NCC = normative commitment to change; UTI = using telemedicine intention; ATUT = attitude towards using telemedicine; SN = subjective norms.

Based on the SEM test that has been conducted, several variables and items need to be correlated to get a fit model. The correlated variables include readiness for change with cognitive experience state and cognitive experience state and continuance commitment to change with a normative commitment to change. In addition, several items need to be eliminated in the readiness for change construct, namely, one item from the participating dimension and one item from the promoting dimension. Similarly, one item was eliminated from the continuance commitment to change construct. Finally, the SEM model was fit with RMSEA = 0.059, RMR = 0.037, CFI = 0.954, IFI = 0.955, and TLI = 0.943. Furthermore, for hypothesis testing, the results of the AMOS 23 output are shown in Figure 4.

The results presented in Study 2 show that readiness for change plays a role in all components of commitment to change, namely, affective commitment to change, continuance commitment to change, and normative commitment to change. In addition, the affective experience state also plays a role in all components of commitment to change. However, it was found that the cognitive experience state does not play a role in all components of commitment to change. Then, Study 2 also obtained results regarding the perceptions and experiences of young people who have used telemedicine. The results are categorized in Table 4.

**Figure 5:**
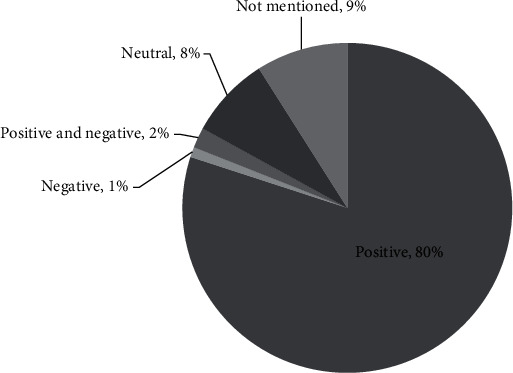
Experience of using telemedicine.

**Figure 6:**
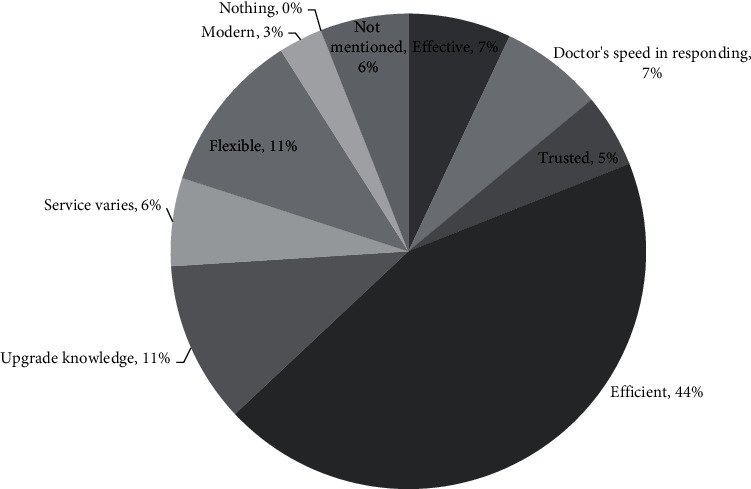
The reason for using telemedicine.

**Figure 7:**
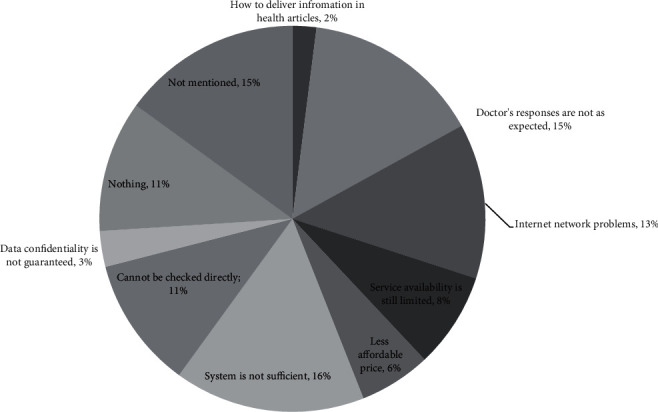
The dislike of telemedicine services.

The experiences of the younger generations who have used telemedicine are varied. In Figure 5, when using telemedicine, 80% had positive experiences, 1% had negative experiences, 2% had both positive and negative experiences, 8% had neutral experiences, and 9% did not answer (see Figure 5). The things they like most about using telemedicine services include being efficient (44%), flexible (11%), and adding insight (11%) (see Figure 6). Meanwhile, the things they dislike most about using telemedicine include inadequate systems (16%), doctors' responses that do not meet expectations (15%), and Internet network constraints (13%) (see Figure 7).

**Figure 8:**
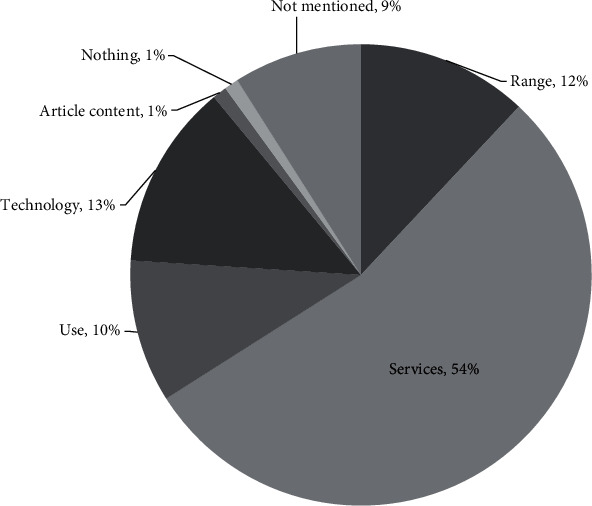
User's expectation of telemedicine.

Young people who have used telemedicine have various expectations for telemedicine facilities in the future. These expectations are grouped into several categories; the most common are related to the need to improve telemedicine services (54%), the expansion of telemedicine services to remote areas (12%), and the hopes to use increasingly sophisticated technology (13%). In addition, participants also hoped that telemedicine could be used for various groups (10%). Finally, a small percentage (1%) of participants emphasized the importance of health articles on telemedicine being written in a clear and concise style to avoid encouraging self-diagnosis (see Figure 8).

## 6. Discussion

This section discusses the results from Study 1 and Study 2. The results of the two studies showed that readiness for change equally plays a positive role in each component of commitment to change, namely, affective commitment to change, continuance commitment to change, and normative commitment to change. Readiness for change is described as the best predictor of attitude towards commitment and support for change [43]. Individuals will not participate in the changes that are/will be implemented or feel any commitment when they perceive themselves as unprepared for the change program [44]. A study by Fowe [45] showed that the readiness for change depends on internal and external factors such as structural characteristics, network, communication, policy, incentives, and patient needs. Furthermore, aside from the organizational context, the user's knowledge and beliefs shape the readiness to change in telehealth/telemedicine [46]. Therefore, internal and external factors could act as opportunities during the development and implementation of telemedicine by improving positive user experience.

This study's findings further revealed that attitude towards telemedicine plays a positive role in the intention to use telemedicine. Utilizing the TPB, this result aligns with research by Degerli and Ozkan-Yildirim [16], which also identified a positive relationship between individuals' attitudes and their intention to use telemedicine. Evaluation of telemedicine use experience contributes most significantly to forming attitudes that directly influence the intention to use it [47]. Supporting this notion, the research by Ahadzadeh, Ong, and Wu [17] demonstrated that an individual's attitude is a mediating factor in examining the relationship between health factors and intention to use health technology/telemedicine. Specifically, the attitude towards telemedicine plays a positive role in the intention to use it. This can be attributed to increased clarity regarding telehealth/telemedicine, especially on privacy matters, leading to greater user acceptance. A study by Rankine et al. [48] provides empirical evidence, showing that some adolescents have been positively accepting telemedicine, especially on confidential matters, while some others express concerns about potential privacy breaches by institutions.

Study 1 revealed that subjective norms do not play a role in the intention to use telemedicine. Previous research supports these findings, where social influence was not associated with the behavioral intention to adopt a telemedicine platform [49]. Similarly, Baudier et al. [50] found that social influence is not a significant factor in technology adoption. The surrounding environment does not easily influence millennials in purchasing because intentions arise from self-awareness, with behavior driven by a desire to achieve specific outcomes [51]. Meanwhile, it is necessary to consider not only perceived social pressure but also feelings of personal moral obligation or responsibility to perform or not perform certain behaviors [52]. Hence, future research can consider the role of perceived behavioral control in the intention to use telemedicine among younger generations who have never used telemedicine.

Affective commitment to change, continuance commitment to change, and normative commitment to change were found to have no role in telemedicine intention. This suggests that among younger generations who have never used telemedicine, commitment of changing from face-to-face healthcare to telemedicine services will not be affected by the intensity of their telemedicine use. This is likely because the behavioral context of the two variables is different. While commitment to change explains concepts related to individual conditions to change, intention explains more about the concept of using telemedicine. Huang and Cheng [31] found a significant relationship between commitment and intention when both constructs targeted the same behavioral concept, namely, commitment to learning sustainability. In addition, the three components of commitment to change function as predictors for change-support behavior [37]. In line with Hill et al. [53], commitment to change is crucial in encouraging individuals to support the change program. Therefore, commitment to change will more likely play a role in change-supportive intention or change-support behavior.

Study 2 revealed that one component of the online customer experience (affective experience state) plays a role in commitment to change. This finding can be explained using the social exchange theory, which has been adopted to explain customer engagement and closeness in an industrial buyer–seller relationship [54]. This theory explains that service providers and customers can develop a sense of shared responsibility for service arrangements, ultimately shaping customer perceptions of service outcomes [55]. Concerning social exchange theory, another study by Abbas et al. [56] has supported this argument by showing that social media could facilitate the learning process that leads to behavioral change and readiness to change by raising knowledge and awareness through social interaction or social exchange. The positive experience felt when using telemedicine will form customer loyalty to telemedicine in the future [37]. Positive experiences can also influence the commitment to change because they could shape people's perspective to accept the change [57]. In relation to the social exchange theory, the study by Sulaiman et al. [54] suggests that the more customers experience a greater return from their respective branded products, the more they are likely to be committed to the products [54]. However, a previous study highlights the need for multifactorial strategies encompassing culture, environment, and effectiveness to give a positive online customer experience [58]. Therefore, this study conceptualizes that the experiences formed from the cognitive and affective aspects of the younger generation when using telemedicine will affect their behavior, which, in this case, is the commitment to change.

Studies on healthcare have demonstrated that the social exchange between physicians and patients regarding information behaviors in online health communities can improve patient compliance [59]. Similarly, studies applying the social exchange theory have emphasized the relationship between the patients and the healthcare staff, leading to the adoption of patients on telehealth/telemedicine systems [60, 61]. However, it should be noted that there are rules that should be followed. Cropanzano and Mitchell [62] argue that the rules of social exchange theory have been emphasized more on the reciprocity principle and the type of transaction in a social relationship. As the social exchange theory posits, social behavior results from an exchange process [54]. The purpose of this exchange is to maximize benefits and minimize costs. Through this lens, young generations weigh telemedicine's potential benefits and risks.

The findings from Study 2 suggest that young people's affective experiences with telemedicine will lead to their commitment to change. The social exchange theory explains that affective experience is an exchange process that produces commitment to change as a behavior. This aligns with prior research demonstrating that positive affective feelings about telemedicine significantly contribute to the intention to use it [17]. The affective experience state positively affects continuance commitment to change and normative commitment to change. However, it has a negative effect on affective commitment to change. In other words, the young generation's experiences shape their perceptions of telemedicine as beneficial (continuance) and necessary/obligatory (normative). Meanwhile, a positive affective state does not necessarily translate to a stronger desire to use telemedicine. Individuals will commit to change if they perceive it as both beneficial and obligatory [35, 63]. Therefore, the higher the affective experience state, the lower their affective commitment to change. This explanation concludes that the younger generation will commit to change towards telemedicine rather than face-to-face healthcare because they need telemedicine, not just because they want it.

One potential explanation for the negative relationship between affective experience state and affective commitment to change lies in the inherent differences between product categories searched on online sites. Yoon [64] explains that different product categories lead to different customer practical experiences. The different atmospheres formed by online services may not involve affective aspects. However, telemedicine is different from online purchasing. Telemedicine will provide a more engaging experience than online purchases because most telemedicine services involve a consultation process involving the doctor and the customer. In addition, telemedicine is a health service that individuals use when they need help, whereas online stores are only used because people want to buy something. Therefore, based on the findings of this study, the younger generation will experience an affective state where they feel the need and necessity to use telemedicine rather than pure desire. Improving user performance and effort expectancy can enhance user confidence in telemedicine applications [65]. It is important to acknowledge that adolescents possess a strong understanding of technologies and a good awareness of health issues. Therefore, user performance may hold greater significance in fulfilling broader needs beyond telemedicine use, given the younger generation's existing technological proficiency and health awareness [66, 67].

The cognitive experience state was found to have no role in all components of commitment to change. This result differs from Farida and Roesman's [42] research, which suggests that millennials who belong to the younger generation are more interested in cognitive experiences than affective experiences in online purchases. Feelings of trust and satisfaction will influence individuals' loyalty to online services [39]. Those feelings are individuals' affective states. Thus, it can be concluded that affective experience states can better influence individuals' loyalty or commitment to online services. Previous studies have proven that reliability, trust, and satisfaction become significant variables that could affect users' commitment to the application [68]. Application stimuli can arouse customers' emotions, influencing their decision-making perceptions, evaluations, and cognition [69]. Consequently, the affective experiences gained by the younger generations when using telemedicine will further influence their behavior, encouraging them to adopt telemedicine as an alternative to traditional face-to-face healthcare delivery.

The younger generations who have used telemedicine will undoubtedly be able to assess and perceive how the telemedicine service is used and whether it has provided an enjoyable experience. Properly analyzing the needs of patients is necessary to increase their involvement in telemedicine [70]. In Study 2, it was found that most of the younger generations already had a positive experience when using telemedicine. They liked telemedicine because it was more efficient and flexible, and it gave them more insight into their health needs [5]. However, the things that are most disliked by them are the inadequate systems, doctors' responses that are not as expected, and Internet network constraints. This aligns with Acharya and Rai [3] who explained that the challenges in using telemedicine are matters related to technical issues, communication with doctors, and arranging schedules for consultations. Beyond these concerns, the young generation in this study hopes that telemedicine healthcare services can provide even better services by expanding their reach to remote areas and incorporating more sophisticated technology to improve usability and ease of access. Additionally, they also emphasize that telemedicine could be used by various groups and provide knowledge about health through adequate health articles.

Factors such as readiness for change and affective experiences are essential for a successful transition to telemedicine use. To increase the use of telemedicine among the younger generations, increasing individual readiness, enhancing adaptability, and developing the quality of telemedicine are essential. This evaluation is necessary because a study stated that the failure rate in implementing telemedicine was 70% [14]. In addition to encouraging participation in telemedicine, the evaluation can also be used as a guide to improve telemedicine for its users. In addition, this research shows that positive perceptions of telemedicine play an essential role in increasing the willingness to adopt telemedicine among younger generations who have never used it. Thus, this research recommends effective telemedicine marketing strategies and continuous quality improvement efforts of telemedicine services to increase positive impressions and trust, ultimately leading to increased utilization of telemedicine among the younger generation.

### 6.1. Theoretical Implication

This section explains the implication of the theory used in Study 1 and Study 2. This study contributes to strengthening the theory of readiness for change and commitment to change in the context of telemedicine adoption. It can be explained by the TPB, which defines preparedness for change as an attitude and commitment to change as an intention. The best predictor of attitude towards commitment and support for change is stated as readiness for change. Findings confirmed that readiness predicts significant positive improvements in commitment. Individuals will only participate in ongoing changes or feel committed to them when they feel ready to participate in the change program. This means that the commitment relied on the readiness itself. It is important for this study to acknowledge the internal and external factors shaping the readiness for change. Therefore, when internal and external factors are involved in shaping readiness, it could lead to a positive attitude that accepts such changes in society. The change in society has accelerated considerably, especially since the COVID-19 pandemic made a massive impact on all individuals. Furthermore, the readiness for change becomes a vital factor in the attitudes and acceptance of change. This study further strengthens the argument of the TPB, which states that attitude towards using telemedicine increases intention to use it. Thus, attitude is essential to improve intention.

### 6.2. Policy Recommendation

This section will explain several policy recommendations from Study 1 and Study 2 in this research. To ensure more significant telemedicine equity, policy changes should address barriers that disproportionately affect marginalized patient populations and those who serve them [68]. This research provides several practical recommendations for policymakers, medical practitioners, and stakeholders to increase the use of telemedicine, especially for the younger generation. First, there are suggestions for young people who have never utilized telemedicine to strive for positive attitudes towards telemedicine behavior. This can be accomplished by expanding the promotion of telemedicine healthcare services with suitable facilities. Second, promoting the emotional experience that incorporates the customer's emotions when using telemedicine can foster a sense of benefit and necessity. This will affect their commitment to adopt telemedicine healthcare services because they believe they will benefit and make telemedicine necessary in the current situation. Third, it is imperative for stakeholders to actively address the concern of privacy breaches in telemedicine, instilling confidence in individuals and alleviating any apprehensions they may have regarding privacy issues associated with telemedicine. Finally, it is essential to examine technical factors, such as the convenience of accessing telemedicine to enhance its usability and influence its purpose.

## 7. Implications

The implications of Study 1 and Study 2 will be discussed in this section. Based on both Study 1 and Study 2, most of the young people who have used telemedicine had a pleasant experience. They believe telemedicine is more efficient and adaptable and could broaden their perspectives. However, the younger generations perceive the telemedicine system in Indonesia as insufficient, with doctors' online communication failing to meet their expectations. Privacy has also become an essential aspect of telemedicine, with fear of privacy breaches since health data has become personal and confidential to individuals. In addition, they are also constrained by the Internet network when using telemedicine. Therefore, the younger generation in this study indicated the need for increased telemedicine services through more advanced technology. The need to expand coverage to remote areas, increase data security, and increase the availability of relevant health resources are also in demand. They emphasized the need for ongoing improvements on telemedicine service quality and functionality.

## 8. Conclusions

In this section, this research concludes with the findings based on the results from Study 1 and Study 2. According to the findings of this study, when young generations who have never used telemedicine or who have used telemedicine have a readiness for change, their commitment to change increases. The affective commitment to change, continuous commitment to change, and normative commitment to change that people feel demonstrate their commitment to change. However, all components of commitment to change and subjective norms do not influence the intention to use telemedicine among younger generations who have never used it. In other words, their dedication to shifting healthcare delivery towards telemedicine and the encouragement from others do not exert a significant influence on their decision to utilize telemedicine. However, their attitude regarding telemedicine substantially impacts their intention to use telemedicine. Younger generations who have never utilized telemedicine will decide if it is a positive behavior, neither because of social influence nor their commitment to change. The more individuals regard the activity as favorable, the more likely they are to use telemedicine.

The younger generations who already utilize telemedicine perceive it differently. The influence of affective experience on telemedicine applications is enormous, while the cognitive experience does not significantly influence it. However, their emotional experiences will reduce commitment to changes driven solely by their desires. Individuals who experience more extraordinary positive emotions are more likely to accept the change because they view it as a lost opportunity if they do not switch to telemedicine services.

Telemedicine has become a necessity during and after the pandemic. However, there are advantages and disadvantages to this. Therefore, telemedicine service providers must focus on strategies to maintain the user engagement of telemedicine, ensuring its acceptance in society. This study collected data regarding intentions to utilize telemedicine and the level of commitment to adopt telemedicine among the younger generations. By identifying many factors that may influence telemedicine services, the findings have provided valuable insights for society, healthcare providers, and health practitioners, particularly regarding the specific needs of young people.

## 9. Limitation and Future Research

This study contains various limitations that must be considered for future research initiatives. First, this study was undertaken in the setting of a pandemic so that future research in the postpandemic period can provide an overview of the characteristics that influence the use of telemedicine in everyday situations. Second, while this study focuses on the younger generation as research respondents, future research may explore the younger and the preceding generations to acquire a more comprehensive picture and compare telemedicine use across generations. Third, this study used a cross-sectional strategy with one-time data collection. Longitudinal studies can be conducted to assess the long-term impact of telemedicine adoption on healthcare outcomes and patient satisfaction. However, mixed methods could also be conducted to examine the impact of telemedicine adoption and, more specifically, the effectiveness and efficiency of telemedicine implementation on patients. Fourth, this study's respondents are young individuals from one city in Indonesia. For generalization, it is necessary to expand the reach of the representation to include younger generations in other regions of Indonesia.

## Figures and Tables

**Table 1:** Instrument test results in Study 1.

**Instrument name**	**Adapted instrument**	**Internal consistency value (**∝**)**	**GFI**	**CFI**	**NFI**	**IFI**	**RMSEA**	**Factor loading**
Readiness for change	Parahyanti [39], an adaptation of the original of Hanpachern, Morgan, and Griego [40]	0.919	0.941	0.947	0.954	0.975	0.092	0.692–0.874
Affective commitment to change	Muasaroh [41], an adaptation of the original of Herscovitch and Meyer [35]	0.804	1.000	1.000	1.000	1.000	1.000	0.675–0.815
Continuance commitment to change	Muasaroh [41], an adaptation of the original of Herscovitch and Meyer [35]	0.814	1.000	1.000	1.000	1.000	0.000	0.555–0.980
Normative commitment to change	Muasaroh [41], an adaptation of the original of Herscovitch and Meyer [35]	0.750	1.000	1.000	1.000	1.000	0.000	0.461–0.968
Using telemedicine intention	Widyapraba, Susanti, and Herdiyanti [33]	0.872	1.000	1.000	1.000	1.000	0.000	0.785–0.882
Attitude towards using telemedicine	Widyapraba, Susanti, and Herdiyanti [33]	0.838	1.000	1.000	1.000	1.000	0.000	0.750–0.875
Subjective norms	Widyapraba. Susanti, and Herdiyanti [33]	0.860	1.000	1.000	1.000	1.000	0.000	0.791–0.845

**Table 2:** The structural equation modeling result of Study 1.

**Variable**	**Estimate**	**SE**	**p** ** value**⁣^∗^	**Explanation**
RFC ➔ ACC	0.832	0.102	< 0.01	Hypothesis 1 accepted
RFC ➔ CCC	0.712	0.134	< 0.01	Hypothesis 2 accepted
RFC ➔ NCC	0.710	0.119	< 0.01	Hypothesis 3 accepted
ACC ➔ UTI	0.138	0.169	0.412	Hypothesis 4 rejected
CCC ➔ UTI	−0.177	0.091	0.052	Hypothesis 5 rejected
NCC ➔ UTI	0.156	0.097	0.108	Hypothesis 6 rejected
ATUT ➔ UTI	1.296	0.458	0.005	Hypothesis 7 accepted
SN ➔ UTI	−0.394	0.269	0.143	Hypothesis 8 rejected

⁣^∗^*p* value is significant if < 0.05.

**Table 3:** Instrument test results in Study 2.

**Instrument name**	**Adapted instrument**	**Internal consistency value (**∝**)**	**GFI**	**CFI**	**NFI**	**IFI**	**RMSEA**	**Factor loading**
Readiness for change	Parahyanti [39], an adaptation of the original of Hanpachern, Morgan, and Griego [40]	0.922	0.960	0.986	0.969	0.986	0.067	0.724–0.861
Affective commitment to change	Muasaroh [41], an adaptation of the original of Herscovitch and Meyer [35]	0.824	1.000	1.000	1.000	1.000	1.000	0.771–0.791
Continuance commitment to change	Muasaroh [41], an adaptation of the original of Herscovitch and Meyer [35]	0.687	1.000	1.000	1.000	1.000	0.000	0.439–0.777
Normative commitment to change	Muasaroh [41], an adaptation of the original of Herscovitch and Meyer [35]	0.714	1.000	1.000	1.000	1.000	0.000	0.448–0.947
Cognitive experience state	Farida and Roesman [42]	0.852	1.000	1.000	1.000	1.000	0.000	0.672–0.927
Affective experience state	Farida and Roesman [42]	0.896	1.000	1.000	1.000	1.000	0.000	0.779–0.926

**Table 4:** Structural equation modeling result of Study 2.

**Variable**	**Estimate**	**S.E**	**p** ** value**⁣^∗^	**Explanation**
RFC ➔ ACC	0.542	0.103	< 0.01	Hypothesis 9 accepted
RFC ➔ CCC	0.410	0.167	0.014	Hypothesis 10 accepted
RFC ➔ NCC	0.398	0.104	< 0.01	Hypothesis 11 accepted
CES ➔ ACC	0.203	0.112	0.071	Hypothesis 12 rejected
CES ➔ CCC	−0.169	0.202	0.405	Hypothesis 13 rejected
CES ➔ NCC	−0.134	0.096	0.163	Hypothesis 14 rejected
AES ➔ ACC	−0.116	0.053	0.030	Hypothesis 15 accepted
AES ➔ CCC	0.304	0.095	0.001	Hypothesis 16 accepted
AES ➔ NCC	0.143	0.048	0.003	Hypothesis 17 accepted

⁣^∗^*p* value is significant if < 0.05.

## Data Availability

The datasets used and analyzed during the current study are available from the corresponding author on reasonable request.
